# Antidiabetic Potency, Antioxidant Effects, and Mode of Actions of *Citrus reticulata* Fruit Peel Hydroethanolic Extract, Hesperidin, and Quercetin in Nicotinamide/Streptozotocin-Induced Wistar Diabetic Rats

**DOI:** 10.1155/2020/1730492

**Published:** 2020-06-20

**Authors:** Alaa M. Ali, Mohamed Abdel Gabbar, Sanaa M. Abdel-Twab, Eman M. Fahmy, Hossam Ebaid, Ibrahim M. Alhazza, Osama M. Ahmed

**Affiliations:** ^1^Physiology Division, Zoology Department, Faculty of Science, Beni-Suef University, Beni-Suef, P. O. Box 62521, Egypt; ^2^Biochemistry Department, Faculty of Science, Beni-Suef University, Beni-Suef, P. O. Box 62521, Egypt; ^3^Department of Internal Medicine, Faculty of Medicine, Helwan University, Egypt; ^4^Department of Zoology, College of Science, King Saud University, P. O. Box 62521, Riyadh 11451, Saudi Arabia; ^5^Department of Zoology, Faculty of Science, El-Minia University, P.O. Box 61519, Minya, Egypt

## Abstract

This study is aimed at assessing the antihyperglycemic, antihyperlipidemic, and antioxidant effects of *Citrus reticulata* (*C. reticulata*) fruit peel hydroethanolic extract and two flavonoids, hesperidin and quercetin, in nicotinamide (NA)/streptozotocin- (STZ-) induced type 2 diabetic rats. In addition, GC-MS and HPLC-MS analyses of the extract were performed and the results indicated the presence of multiple flavonoids including hesperidin, quercetin, naringin, and polymethoxylated flavones (nobiletin and tangeretin). To achieve the aim of the study, diabetic rats with NA/STZ-induced T2DM were orally treated with *C. reticulata* fruit peel hydroethanolic extract, hesperidin, and quercetin at a dose of 100 mg/kg b.w./day for four weeks. The treatments with *C. reticulata* fruit peel extract, hesperidin, and quercetin significantly ameliorated the impaired oral glucose tolerance; the elevated serum fructosamine level; the diminished serum insulin and C-peptide levels; the altered HOMA-IR, HOMA-IS, and HOMA-*β* cell function; the decreased liver glycogen content; the increased liver glucose-6-phosphatase and glycogen phosphorylase activities; the deleteriously affected serum lipid profile; the elevated serum AST and ALT activities; and the raised serum creatinine and urea levels in the diabetic rats. The treatments also produced remarkable improvement in the antioxidant defense system manifested by a decrease in the elevated liver lipid peroxidation and an increase in the lowered glutathione content and GPx, GST, and SOD activities. Furthermore, the three treatments enhanced the mRNA expression of GLUT-4 and the insulin receptor *β*-subunit, but only quercetin produced a significant increase in the expression of adiponectin in adipose tissue of diabetic rats. In conclusion, *C. reticulata* fruit peel hydroethanolic extract, hesperidin, and quercetin have potent antidiabetic effects which may be mediated through their insulinotropic effects and insulin-sensitizing actions. In addition, the alleviation of the antioxidant defense system by the extract, hesperidin, and naringin may have an important action to enhance the antidiabetic actions and to improve liver and kidney functions in NA/STZ-induced diabetic rats.

## 1. Introduction

Diabetes mellitus (DM), one of the most common diseases in the world, results from impairments in insulin secretion and/or insulin action leading to disturbances in the metabolism of carbohydrates, lipids, and proteins [1, 2]. The American Diabetes Association (ADA) has classified DM into type 1 DM (T1DM), type 2 DM (T2DM), gestational DM (GDM), and many other specific types of diabetes [3]. T2DM is much more prevalent in humans than T1DM and is responsible for 90% of DM incidence [4, 5]. The main reasons for T2DM are impaired tissue insulin sensitivity and insulin resistance which was coupled to pancreatic *β*-cell dysfunction [6–8]. Many experimental animal models of T2DM were applied by several publications to validate the use of new therapies and to elucidate the underlying molecular mechanism(s) of action of the tested drugs [9, 10].

Nicotinamide (NA)/streptozotocin- (STZ-) induced DM is the most commonly used animal model of T2DM in rats. STZ, an antibiotic drug formed by *Streptomyces achromogenes*, has damaging effects on the *β*-cells in the islets of Langerhans [11–13]. Many reports stated that the damaging effect of STZ on *β*-cells of pancreatic islets is caused by the stimulation of oxidative stress and suppression of antioxidant defense [14–16]. Furthermore, the intracellular biotransformation of STZ results in the production of nitric oxide (NO) which speeds up the formation of DNA strand breaks, leading to *β*-cells' necrosis [17]. NA injection before STZ in this DM-induced model, on the other hand, partially counteracts the destructive effect of STZ on *β*-cells, and it leads to the loss of the early phase of glucose stimulation of insulin secretion which is a feature of T2DM [18–20]. It was also proven by many investigators that in NA/STZ-induced DM, there are both impairment in insulin secretion and insulin resistance, which is a characteristic feature of T2DM [21–23].

The search for suitable antihyperglycemic agents from natural sources has been focused on plants applied in traditional medicines partly because they have lower side effects than the currently used conventional drugs [24]. Recently, there is an increased interest in the medical benefits of flavonoids because their supplementation seems to be accompanied by reduced risks for certain severe maladies and increased survival as stated by previous publications [25–27]. Citrus fruit peels, i.e., the outer layers of many fruits including lemons, oranges, mandarins, and grapefruits, have been demonstrated to be rich in flavonoid content [27–30]. Flavonoids found in citrus fruits were mainly allocated to three classes: flavanones, flavones, and flavonols [31].

*Citrus reticulata* (*C. reticulata*) or tangerine fruit peels have been shown to contain high concentrations of three flavanones: hesperidin, naringin, and narirutin [32]. Citrus peel also contains good quantities of flavonol and quercetin [33]. Hesperidin, a glycosylated flavanone of hesperetin, has been reported by Constantin et al. [34] and Parhiz et al. [35] to decrease intestinal glucose absorption and inhibit the gluconeogenic pathways, thereby leading to antihyperglycemic actions in diabetic human beings. Quercetin, a principal flavonol found in citrus fruits especially in fruit peels, was found to have antidiabetic actions in diabetic animal models at doses of 10, 25, and 50 mg/kilogram body weight (kg b.w.) [36]. It is a glycone of rutin, and it is a parent compound of a number of various flavonoids [37, 38]. Although the antidiabetic effects of hesperidin and quercetin were reported by some publications, the mechanisms of their antidiabetic actions are not fully elucidated. In addition, further investigations are needed to assess their comparative effects with the crude extract of *C. reticulata* fruit peel.

Therefore, the present study was conducted to assess the comparative antihyperglycemic, antihyperlipidemic, and antioxidant effects of *C. reticulata* fruit peel hydroethanolic extract, hesperidin, and quercetin in NA/STZ-induced DM in Wistar rats and to suggest their mechanisms of action.

## 2. Materials and Methods

### 2.1. Experimental Animals

Adult male rats of Wistar strain weighing about 130-150 g and aged 10-12 weeks were used in the present experimental research work. The animals were supplied from the animal house of the National Research Center (NRC), El-Tahrir Street, Dokki, Giza, Egypt. They were maintained under strict care for about 10 days before the start of the experiment to exclude any intercurrent infection. The rats were housed in clean polypropylene cages (six rats/cage) with a well-aerated standard stainless steel frame and wood mulch at the bottom of cage. The rats were maintained under normal controlled atmospheric temperature (25 ± 5°C), humidity (55 ± 5.6), and daily normal 12-hour (hr) light/dark cycle. Moreover, they had free access to water and were provided daily with standard pelleted chow diet *ad libitum*. All animal procedures were in accordance with the guidelines and recommendations of the Experimental Animal Ethics Committee for Use and Care of Animals, Faculty of Science, Beni-Suef University, Egypt (ethical approval number is BSU/FS/2015/14**)**. All instructions were followed, and all precautions were considered to minimize discomfort, distress, and pain of rats under investigations.

### 2.2. Drugs and Chemicals

STZ (2-deoxy-2-(3-methyl-3-nitrosoureido)-D-glycopyranoside), NA, hesperidin, and quercetin were obtained from Sigma-Aldrich Chemical Company, St. Louis, MO, USA. NA, hesperidin, and quercetin were kept at 2–4°C, while STZ was stored at -20°C. All other used chemicals were of analytical grade and were commercially obtained.

### 2.3. Extract Preparation of *C. reticulata* Fruit Peel

*C. reticulata* or tangerine fruits were purchased from the local market at Beni-Suef Governorate, Egypt. The purchased fruits were manually flaked and were cleaned by washing with running water till completely clean. The washed peels were then dried in a good aerated area. Then, the dried peels were ground to a powder by an electric mortar. The finely obtained powder (0.5 kg) was drenched in 70% ethanol for 3 days. The mixture was filtered by using a Whatman No. 2 filter paper for removal of peel particles. The water and ethanol were vaporized by a Rotavapor to obtain a semisolid viscous mass which was stored in dark bottles in a deep freezer at -30°C pending its use for the treatment.

### 2.4. Gas Chromatography-Mass Spectrometry (GC-MS) Analysis

Chemical analysis of *C. reticulata* peel hydroethanolic extract was performed in the Central Laboratory of the Faculty of Postgraduate Studies for Advanced Sciences, Beni-Suef University, Egypt, by using the Gas Chromatography (GC) System 7890A/5975C Inert Mass Spectrometry (MS) with a Triple Axis Detector, Agilent Technologies, Germany. The constituents were recognized by comparing their mass spectra with the spectra of derivatives in the Library Search Report (C:\Database\NIST11.L; C:\Database\demo.l) as well as in the NIST08s, WILEY8, and FAME libraries.

### 2.5. High-Performance Liquid Chromatography- (HPLC-) Mass Spectrometry (MS) Analysis

HPLC-MS analysis of *C. reticulata* fruit peel hydroethanolic extract was performed in the Central Laboratory of the Faculty of Postgraduate Studies for Advanced Sciences, Beni-Suef University, Egypt, by using the HPLC-MS system, 1260 Infinity, Agilent Technologies, Germany coupled with a Diode Array Detector (DAD). Standards including gallic acid, naringin, quercetin, hesperidin, nobiletin, and tangeretin were used to identify their peaks in the HPLC-MS chromatogram. The *C. reticulata* fruit peel hydroethanolic extract was dissolved in water : methanol (80 : 20 *v*/*v*) at a concentration of 10 mg/3 ml and filtered with a 0.45 *μ*m filter, before injection of 20 *μ*l into the HPLC system. Spectral UV data from all peaks were collected in the range of 240-400 nm, and chromatograms were recorded at 340 and 270 nm according to the method of Negri et al. [39].

### 2.6. Induction of T2DM

Experimental T2DM was induced in male Wistar rats, fasted for 16 hours (hrs), by a single intraperitoneal (i.p.) injection of STZ at a dose level of 50 mg STZ/kg b.w. (dissolved in citrate buffer of pH 4.5), 15 minutes after the i.p. injection of 120 mg NA/kg b.w. [40]. Ten days after NA/STZ injection, overnight-fasted rats were orally supplemented with glucose (3 g/kg b.w.) by oral gavage. After 2 hrs of oral glucose administration, blood samples taken from the lateral tail vein were left to coagulate and then centrifuged. Thereafter, serum glucose level was detected. After screening of serum glucose levels, the rats which have serum glucose levels of 180-300 mg/dl after 2 hrs of oral glucose loading were considered mildly diabetic and were included in the experiment. Rats with serum glucose levels outside this range were excluded.

### 2.7. Experimental Design

The rats included in the experiment were divided into five groups, each group comprising six rats as follows:

1. *Group 1* was regarded as the normal control group and received the equivalent volume of the vehicle, 1% carboxymethyl cellulose (CMC), by oral gavage daily for four weeks
2. *Group 2* was regarded as the diabetic control group and received the equivalent volume of 1% CMC by oral gavage daily for four weeks
3. *Group 3* served as diabetic rats that were treated with *C. reticulata* fruit peel hydroethanolic extract at a dose level of 100 mg in 5 ml 1% CMC/kg b.w./day [41], by oral gavage, for four weeks
4. *Group 4* served as diabetic rats that were treated with hesperidin (Sigma-Aldrich Chemical Company, MO, USA), at a dose level of 100 mg (dissolved in 5 ml 1% CMC)/kg b.w./day, by oral gavage, for four weeks [42].
5. *Group 5* served as diabetic rats that were treated with quercetin (Sigma-Aldrich Chemical Company, MO, USA), at a dose level of 100 mg (dissolved in 5 ml 1% CMC)/kg b.w./day, by oral gavage, for four weeks [43].

Each week, the dose was adjusted according to the alterations in b.w. to stabilize the correct dose per kg b.w. of rats during the entire period of the experiment.

### 2.8. Blood and Tissue Sampling

At the day before decapitation, an oral glucose tolerance test (OGTT) was performed for all individual rats. Blood samples were withdrawn from the lateral tail veins of overnight-fasted rats at 30, 60, 90, and 120 minutes following the oral glucose loading (3 g/kg b.w.), left to clot, and centrifuged at 4000 rounds per minute (rpm) for 15 minutes. After that, sera were quickly collected for the detection of serum glucose levels.

A day following OGTT, the overnight-fasted animals were anaesthetized by diethyl ether inhalation anaesthesia and blood samples were obtained from the jugular vein. Then, after decapitation by cervical dislocation, rats were dissected and liver, visceral adipose tissue, and pancreas were excised and perfused in saline. The blood from each rat was collected in gel and clot activator tubes and centrifuged at 4000 rpm for 15 minutes. The obtained sera were stored in a deep freezer at -30°C until they were used for biochemical detection. The liver was kept in a deep freezer at -30°C pending its use for the determination of liver glycogen content and homogenization in saline (2% *w*/*v*). Pieces of visceral adipose tissue (3 mm^3^) were kept in a deep freezer at -70°C pending their use in RNA isolation and RT-PCR analysis. Pancreas from each rat was fixed in 10% neutral buffered formalin and transferred to the Pathology Department, National Cancer Institute (NCI), Cairo University, Cairo, Egypt, for processing, blocking in wax, sectioning, and staining with the trichrome PAS method.

### 2.9. Biochemical Assays

Serum glucose level was determined based on the method of Trinder [44] by a reagent kit obtained from Randox Laboratories, United Kingdom (UK). Serum fructosamine level was determined according to the method of Baker et al. [45]. Serum insulin and C-peptide levels were determined by an ELISA kit obtained from Linco Research Inc., USA, according to the manufacturer's instruction. Homeostasis model assessment of insulin resistance (HOMA-IR), homeostasis model assessment of insulin sensitivity (HOMA-IS), and homeostasis model assessment of *β*-cell function (HOMA-*β* cell function) were calculated according to the equations described by Mishra et al. [46] and Aref et al. [47]. The measurement of serum total cholesterol (TC) and HDL-cholesterol (HDL-C) levels was performed based on the publication of Allain et al. [48], using a reagent kit obtained from Randox Laboratories (UK). Serum triglyceride (TG) level was determined based on the method of Finley and Tietz [49]. Serum LDL-cholesterol (LDL-C) level was determined based on Friedewald et al.'s [50] formula (LDL − C = TC − HDL − C − TG/5). Serum vLDL-cholesterol (vLDL-C) was calculated based on Norbert's [51] formula (vLDL − C = TG/5). FFA level in serum was estimated based on the publication of Duncombe [52]. Aspartate transaminase (AST) and alanine transaminase (ALT) activities in serum were measured, respectively, based on the publication of Gella et al. [53], by reagent kits delivered from Randox Laboratories (UK). Serum creatinine and urea levels were determined by using kits obtained from Biosystems S.A. (Spain) according to the methods of Fabiny and Ertingshausen [54] and Tabacco et al. [55], respectively.

Liver glycogen content was estimated based on the procedure of Seifter et al. [56]. Glucose-6-phosphatase (G-6-Pase) and glycogen phosphorylase activities in liver homogenates were assayed based on the procedures of Kabir and Begum [57] and Stalmans and Hers [58], respectively.

Liver lipid peroxidation (LPO) was estimated by malondialdehyde (MDA) detection based on the publication of Preuss et al. [59]. Liver glutathione (GSH) content was detected based on the publication of Beutler et al. [60]. Liver glutathione peroxidase (GPx) and glutathione-S-transferase (GST) activities were detected based on the procedures of Matkovics et al. [61] and Mannervik and Gutenberg [62], respectively. The enzyme SOD activity in liver was measured based to the procedure of Marklund and Marklund [63].

### 2.10. Histological Investigation

Pancreatic tissues fixed in 10% neutral buffered formalin were transferred to the Pathology Department, National Cancer Institute, Cairo University, Cairo, Egypt, for processing, which included embedding in paraffin wax, sectioning at 5 *μ*m thickness, and staining with a modified aldehyde fuchsin stain method according to Bancroft and Stevens [64].

### 2.11. RNA Isolation and RT-PCR Analysis

The total RNA was isolated from visceral adipose tissue by the GeneJET RNA Purification Kit obtained from Thermo Scientific Verso 1-Step RT-PCR ReddyMix Kit, Thermo Fisher Scientific Inc., USA according to the publications of Chomzynski and Sacchi [65] and Boom et al. [66]. The levels of isolated RNA were determined and quantified using an ultraviolet (UV) spectrophotometer and taking the absorbances at optical densities (OD) of 260 nm and 280 nm. RNA was quantified and qualified based on Finley and Tietz's [49] formula (RNA (*μ*g/*μ*l) = OD at 260 nm × dilution × 40 *μ*g/ml/1000).

For each extracted RNA sample, the ratio was between OD at 260 nm and OD at 280 nm and the ratio ranged between 1.7 and 2.0 to ensure the high purity of extracted RNA. Thermo Scientific Verso 1-Step RT-PCR ReddyMix was applied for the synthesis of cloned DNA (cDNA) which, in turn, was amplified by using specific forward and reverse primers by 32 Techne thermal cyclers. The primer pair sequences are as follows: GLUT-4—forward: 5′ GCTGTGCCATCTTGATGACGG 3′ and reverse: 5′ TGAAGAAGCCAAGCAGGAGGAC 3′ [1]; insulin receptor *β*-subunit (IR*β*)—forward: 5′ CTGGAGAACTGCTCGGTCATT 3′ and reverse: 5′ GGCCATAGACACGGAAAAGAAG 3′ [67]; adiponectin—forward: 5′ AATCCTGCCCAGTCATGAAG 3′ and reverse: 5′ TCTCCAGGAGTGCCATCTCT 3′ [68, 69]); and *β*-actin—forward: 5′ TCACCCTGAAGTACCCCATGGAG 3′ and reverse: 5′ TTGGCCTTGGGGTTCAGGGGG 3′ [70]).

### 2.12. Statistical Analysis

The obtained individual data were statistically analyzed by one-way analysis of variance (ANOVA) using the PC-STAT program, University of Georgia, followed by the Least Significance Difference (LSD) test to compare various groups with each other [71]. *F*-probability for the detected parameter represents the general effects between groups. All data are represented as mean ± SEM, and significant changes were calculated at *p* < 0.05 and *p* < 0.01 for LSD and at *p* < 0.05, *p* < 0.01, and *p* < 0.01 for *F*-probabilities.

## 3. Results

### 3.1. GC-MS Analysis of *C. reticulata* Peel Hydroethanolic Extract

**Figure 1:**
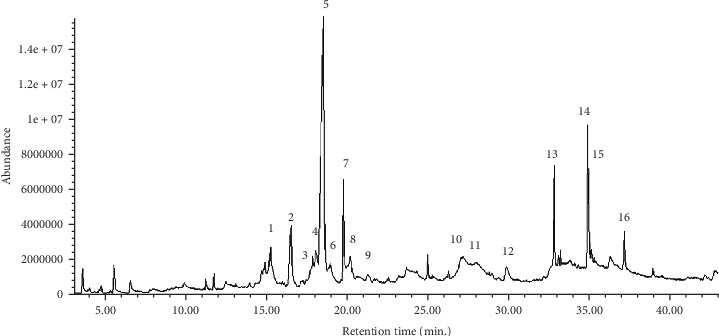
GC-MS chromatogram of *C. reticulata* fruit peel hydroethanolic extract.

The GC-MS analysis (Table 1 and Figure 1) indicated the presence multiple phytochemicals. The main constituents and groups which have a concentration of more than 1% of the total include 4H-pyran-4-one (a cyclic nucleus in the chemical structure of quercetin, naringin, hesperetin, nobiletin tangeretin, etc.), 2,3-dihydro-3,5-dihydroxy-6-methyl-; 5-hydroxymethylfurfural; 4-hexen-3-one, 4,5-dimethyl; phenol, 4-ethyl-; benzaldehyde, 4-hydroxy-; benzaldehyde, 2-hydroxy-; 3,3′,4′,5,5′,7,8-heptamethoxyflavone; 4h-1-benzopyran-4-one, 2-(3,4-dimethoxyphenyl)-5,6,7-trimethoxy-; and *β*-D-glucopyranose, 4-O-*β*-D-galactopyranosyl-; n-hexadecanoic acid; tridecanoic acid; 9,12-octadecadienoic acid (Z,Z)-; (Z)6,(Z)9-pentadecadien-1-ol; 9,12,15-octadecatrien-1-ol, (Z,Z,Z)-; 9,12,15-octadecatrien-1-ol, (Z,Z,Z)-; 9-octadecenamide, (Z)-.

4H-Pyran-4-one, 2,3-dihydro-3,5-dihydroxy-6-methyl-; 5-hydroxymethylfurfural; 4-hexen-3-one, 4,5-dimethyl-; 4h-1-benzopyran-4-one, 2-(3,4-dimethoxyphenyl)-5,6,7-trimethoxy-; and *β*-D-glucopyranose, 4-O-*β*-D-galactopyranosyl- have the highest percent of the total.

### 3.2. HPLC-MS Analysis of Navel Orange Peel Hydroethanolic Extract

**Figure 2:**
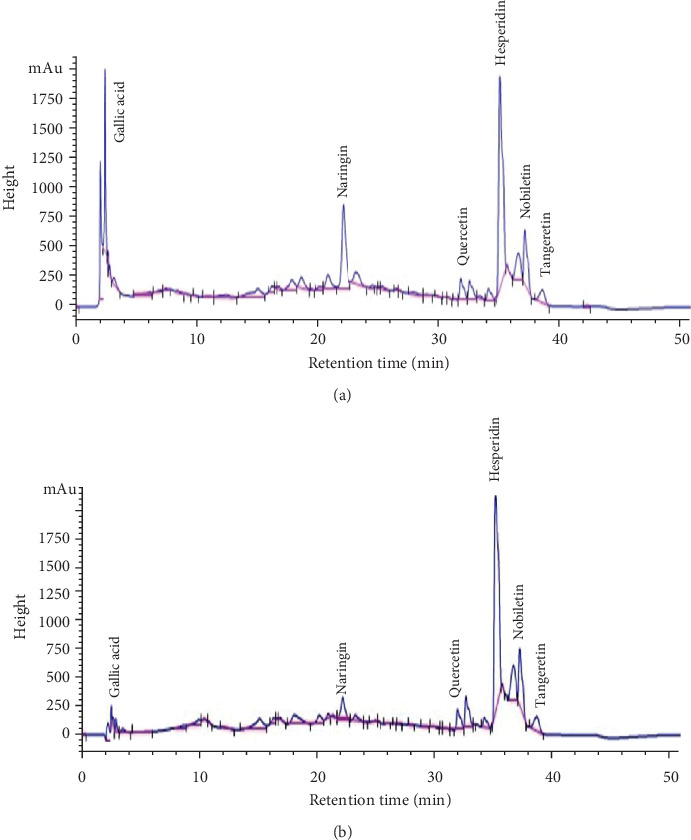
HPLC-MS fingerprint of *C. reticulata* fruit peel hydroethanolic extract at 270 nm (a) and at 340 nm (b) indicating the presence of gallic acid, naringin, quercetin, hesperidin, nobiletin, and tangeretin at abundances (percent of total) of 7.152%, 13.219%, 3.042%, 32.960%, 8.237%, and 2.776% at the signal of 270 nm and 1.036%, 4.403%, 3.018%, 45.018%, 10.095%, and 3.688% at the signal of 340 nm, respectively.

HPLC-MS analysis indicated in Figures 2(a) and 2(b) revealed the presence of gallic acid, naringin, quercetin, hesperidin, nobiletin, and tangeretin at abundances (percent of total) of 7.152%, 13.219%, 3.042%, 32.960%, 8.237%, and 2.776% at a signal of 270 nm and 1.036%, 4.403%, 3.018%, 45.018%, 10.095%, and 3.688% at a signal of 340 nm, respectively. Accordingly, hesperidin, naringin, and nobiletin were the most abundant.

### 3.3. Oral Glucose Tolerance Test (OGTT)

**Figure 3:**
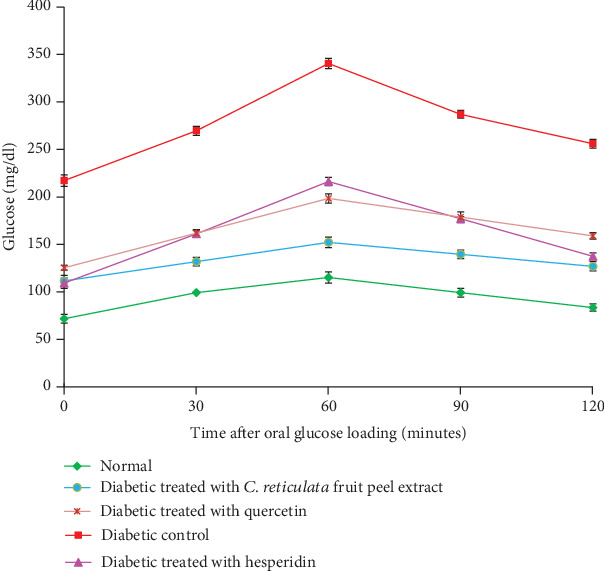
OGT in normal, diabetic control, and diabetic groups treated with *C. reticulata* fruit peel extract, hesperidin, and quercetin. *F*-probability: *p* < 0.001; LSD at the 5% level: 64.80; LSD at the 1% level: 88.30.

Figure 3 showed that the normal animals had a fasting serum glucose level which was significantly lower than that of the diabetic ones. Serum glucose level reached its peak value after 60 minutes following glucose intake (3 g/kg b.w.) and began to decrease during the next 60 minutes to reach its minimum level at 2 hrs of oral glucose loading. On the other hand, the serum glucose level of NA/STZ diabetic control rats also reached its maximum after 60 minutes of oral glucose loading. Then, this value began to decline but in a slower rate and was still higher as compared to the normal one. Furthermore, the diabetic control rats exhibited a highly significant increase in serum glucose level (*p* < 0.01; LSD) as compared to normal ones at all points of OGTT. After 4 weeks of treatment, *C. reticulata* hydroethanolic peel extract, hesperidin, and quercetin produced a highly significant (*p* < 0.01; LSD) hypoglycemic effect on fasting serum glucose level of diabetic treated rats as compared to the nontreated diabetic group. The present data also revealed that hesperidin was the most potent in decreasing the elevated serum glucose levels of NA/STZ-induced diabetic rats at fasting state, while *C. reticulata* hydroethanolic peel extract was the most potent at 30, 60, 90, and 120 minutes after oral glucose administration. As the one-way ANOVA test was applied on OGTT of normal, diabetic control, and diabetic treated rats, the effect between groups on OGTT was very highly significant (*p* < 0.001; *F*-probability).

### 3.4. Effect on Serum Fructosamine Level

**Figure 4:**
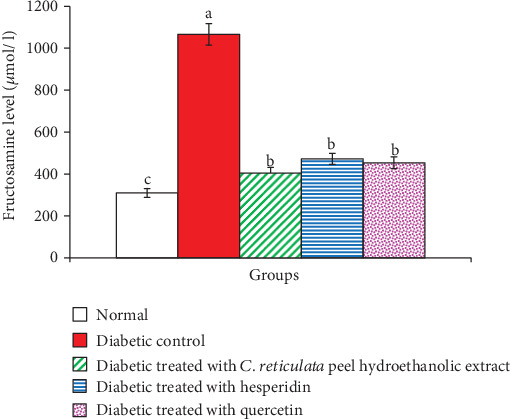
Serum fructosamine level in in normal, diabetic control, and diabetic groups treated with *C. reticulata* fruit peel extract, hesperidin, and quercetin. Means which have different letters are significantly different at *p* < 0.05.

As observed in Figure 4, serum fructosamine level exhibited a significant elevation (*p* < 0.01; LSD) in NA/STZ-induced diabetic animals. The treatment of diabetic rats with *C. reticulata* fruit peel extract, hesperidin, and quercetin induced a significant improvement of the elevated serum fructosamine level recording percentage decreases of -62.11%, -55.74%, and -57.48%, respectively, as compared with the diabetic control rats.

### 3.5. Effect on Serum Insulin and C-Peptide Levels

As shown in Table 2, the serum insulin and C-peptide levels of NA/STZ-induced diabetic animals significantly decreased (*p* < 0.01; LSD); the recorded percentage changes were -51.81% and -66.77%, respectively, as compared to the normal control. The administration of *C. reticulata* fruit peel extract, hesperidin, and quercetin to NA/STZ-induced diabetic rats produced a significant improvement (*p* < 0.01; LSD) of the lowered levels. Hesperidin was the most effective in improving the lowered levels in diabetic rats; the recorded percentage changes were 88.17% and 132.32%, respectively, in comparison with the diabetic control rats. With regard to one-way ANOVA, the general effect on levels of serum insulin and C-peptide between groups was very highly significant (*p* < 0.001; *F*-probability) throughout the experiment.

### 3.6. Effect on Homeostasis Model Assessment of Insulin Resistance (IR), Insulin Sensitivity (IS), and *β*-Cell Function

As represented in Table 3, HOMA-IS and HOMA-*β* cell function of NA/STZ-induced diabetic rats exhibited a highly significant depletion (*p* < 0.01), while the HOMA-IR index exhibited a significant elevation (*p* < 0.01; LSD) in comparison with normal rats; the recorded percentage changes were -54.05%, -73.60%, and 98.88% for HOMA-IS, HOMA-*β* cell function, and HOMA-IR, respectively. The administration of *C. reticulata* fruit peel extract, hesperidin, and quercetin to NA/STZ-induced diabetic rats produced a highly significant improvement of HOMA-IR, HOMA-IS, and HOMA-*β* cell function (*p* < 0.01; LSD) in comparison with the corresponding values of NA/STZ-induced diabetic control. With regard to one-way ANOVA, the general effect between groups was very highly significant since the *F*-probability has *p* < 0.001.

### 3.7. Effects on Liver Glycogen Content and G-6-Pase and Glycogen Phosphorylase Activities

Liver glycogen content exhibited a significant depletion (*p* < 0.01; LSD) in NA/STZ-induced diabetic rats in comparison with the normal group; the recorded percentage decrease was 63.12%. The administration of *C. reticulata* fruit peel extract, hesperidin, and quercetin to NA/STZ-induced diabetic rats caused a significant improvement (*p* < 0.01; LSD) in liver glycogen content. Hesperidin seemed to be the most effective in increasing the liver glycogen content of diabetic rats; the recorded percentage increase was 94.70% in comparison with the diabetic control (Table 4).

Liver G-6-Pase and glycogen phosphorylase activities in the liver of diabetic Wistar rats, on the other hand, exhibited a significant increase (*p* < 0.01; LSD) in comparison with the normal group; the recorded percentage changes were, respectively, 217.20% and 11.02%. The administration of *C. reticulata* fruit peel extract, hesperidin, and quercetin to NA/STZ-induced diabetic rats induced a significant amelioration (*p* < 0.01; LSD) of the raised enzyme activities. With regard to one-way ANOVA, the general effect between groups was very highly significant since the *F*-probability has *p* < 0.001 (Table 4).

### 3.8. Effect on Serum Lipid Profile and FFA Level

The TC, TG, LDL-C, vLDL, and FFA levels (Table 5) showed a significant (*p* < 0.01; LSD) elevation in serum of NA/STZ-induced diabetic rats as compared with the normal group, but the serum HDL-C level exhibited a significant decrease (*p* < 0.01; LSD). The treatment of diabetic rats with fruit peel extract, hesperidin, and quercetin caused a significant improvement (*p* < 0.01; LSD) in TC, TG, HDL-C, LDL-cholesterol, vLDL level, and FFA levels. While hesperidin was the most effective in improving TG, vLDL-C, and FFA levels in diabetic animals, the fruit peel extract was the most effective in amending the deteriorated TC and HDL-C and LDL-C levels (Table 5). With regard to one-way ANOVA, the effect between groups on serum TC, TG, LDL-C, vLDL, and FFA levels was very highly significant since the *F*-probability has *p* < 0.001.

### 3.9. Effect on Serum AST and ALT Activities

The activities of serum AST and ALT in NA/STZ-induced diabetic rats showed a significant elevation (*p* < 0.01; LSD); the recorded percentage changes were 51.23% and 84.33%, respectively. The administration of *C. reticulata* fruit peel extract, hesperidin, and quercetin to NA/STZ-induced diabetic rats resulted in a significant (*p* < 0.001; LSD) improvement of AST and ALT activities. The treatment with hesperidin was the most potent in improving the elevated AST activity. With regard to one-way ANOVA, the general effect on serum AST and ALT activities between groups was very highly significant (*p* < 0.001; *F*-probability) (Table 6).

### 3.10. Effect of Serum Parameters Related to Kidney Functions

Serum creatinine and urea of NA/STZ-induced diabetic rats exhibited a significant increase (*p* < 0.01; LSD) in comparison with the normal control; the recorded percentage increases were 116.11% and 64.78%, respectively. All treatments including *C. reticulata* peel fruit peel extract, hesperidin, and quercetin caused a significant decrease in serum creatinine and urea (*p* < 0.01; LSD) in comparison with diabetic control. The treatment with hesperidin was the most effective in improving the elevated serum creatinine and urea recording percentage decreases of 41.06% and 34.80%, respectively. One-way ANOVA indicated that the effect on serum creatinine and urea between groups was very highly significant (*p* < 0.001; *F*-probability) (Table 7).

### 3.11. Effect on Liver Oxidative Stress and Antioxidant Defense Parameters

The diabetic rats exhibited a significant increase (*p* < 0.01; LSD) in LPO; the recorded percentage change was 126.10% in comparison with the normal group. The treatment of diabetic rats with *C. reticulata* fruit peel hydroethanolic extract, hesperidin, and quercetin produced a significant improvement of the elevated LPO. The three treatments have no significant effects when compared with each other; thus, their effects were more or less similar as compared with the diabetic control (Table 8).

Liver GSH, GPx, GST, and SOD levels significantly decreased (*p* < 0.01; LSD) in the diabetic control group. The treatment of diabetic rats with *C. reticulata* fruit peel extract, hesperidin, and quercetin induced a significant improvement of lowered GSH content, GPx, GST, and SOD activities. The treatment of *C. reticulata* fruit peel hydroethanolic extract was the most effective in increasing GPx activity, while the GSH content and GST and SOD activities were not significant when the three treatments were compared with each other. Regarding one-way ANOVA, the effect between groups was very highly significant since the *F*-probability has *p* < 0.001 (Table 8).

### 3.12. Histological Effects

**Figure 5:**
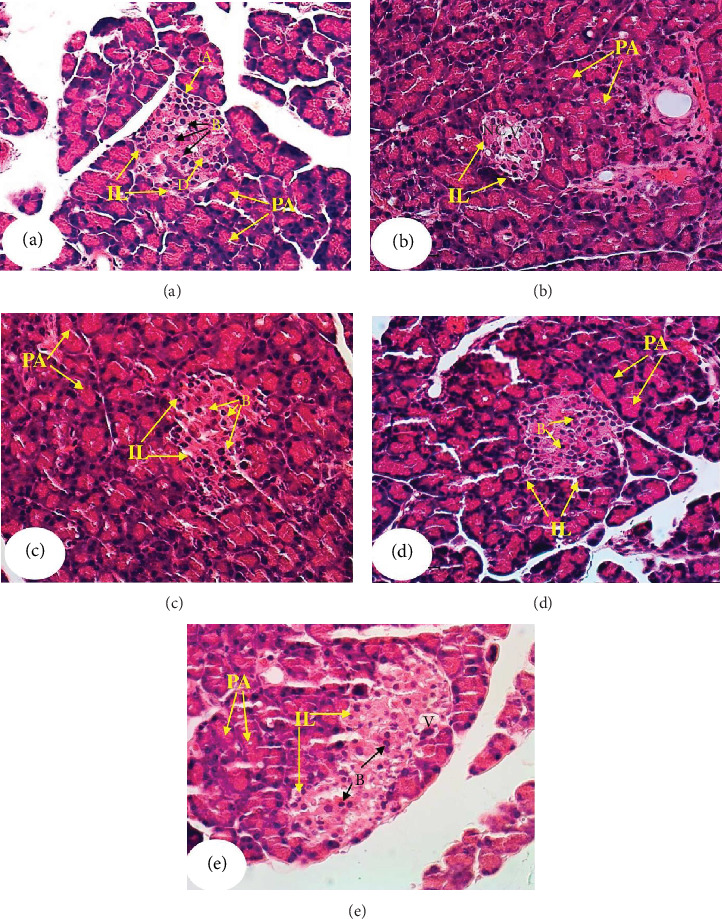
Photomicrographs of pancreata of normal (a), diabetic control (b), and diabetic rats treated with *C. reticulata* fruit peel extract (c), hesperidin (d), and quercetin (e). PA: pancreatic acini; IL: islet of Langerhans; A: alpha cells; B: beta cells; D: delta cells; V: vacuolation; and NC: necrosis.

The pancreas histological changes in NA/STZ-induced diabetic rats and effects of treatments of diabetic rats with *C. reticulata* fruit peel extract, hesperidin, and quercetin are depicted in Figures 5(a)–5(e). The pancreas of normal rats has intact organized pancreatic acini and islets of Langerhans that contain *α*-, *β*-, and *δ*-cells (Figure 5(a)). The diabetic rats showed a decrease in the size of the pancreatic islets, which have a decreased number of islet cells, necrosis, and vacuolations (Figure 5(b)). The treatments of diabetic rats with *C. reticulata* fruit peel extract, hesperidin, and quercetin induced a remarkable improvement in the islets' histological architecture and integrity as observed in Figures 5(c)–5(e), respectively. The treatment with hesperidin seemed to be the most potent in amending the islet histological architecture of diabetic rats. Although the pancreatic islets of diabetic rats treated with quercetin have the largest size, they still exhibited vacuolations and degenerative changes.

### 3.13. Effects on Adipose Tissue mRNA Expressions of Various Genes

**Figure 6:**
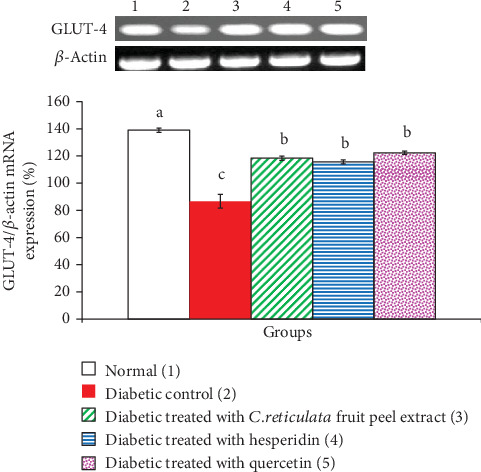
Visceral adipose tissue GLUT-4 mRNA expression relative to *β*-actin in normal, diabetic control, and diabetic groups treated with *C. reticulata* fruit peel extract, hesperidin, and quercetin. Means which have different letters are significantly different at *p* < 0.05.

**Figure 7:**
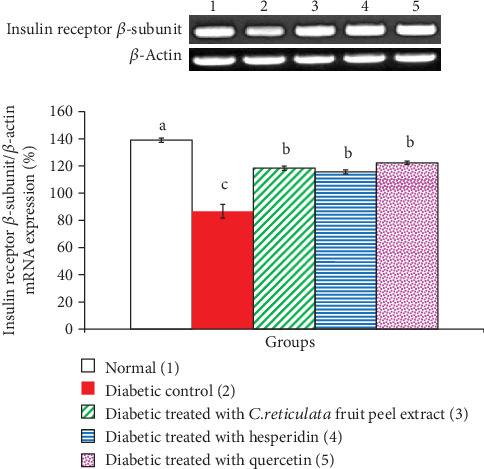
Visceral adipose tissue insulin receptor *β*-subunit mRNA expression relative to *β*-actin in normal, diabetic control, and diabetic groups treated with *C. reticulata* fruit peel extract, hesperidin, and quercetin. Means which have different letters are significantly different at *p* < 0.05.

**Figure 8:**
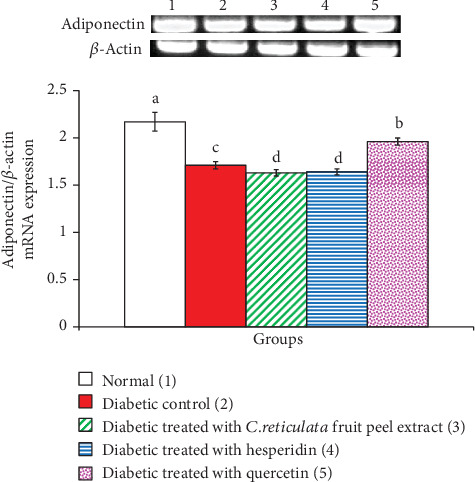
Visceral adipose tissue adiponectin mRNA expression relative to *β*-actin in normal, diabetic control, and diabetic groups treated with *C. reticulata* fruit peel extract, hesperidin, and quercetin. Means which have different letters are significantly different at *p* < 0.05.

*C. reticulata* fruit peel extract, hesperidin, and quercetin effects on adipose tissue GLUT-4, insulin receptor *β*-subunit, and adiponectin mRNA expressions relative to *β*-actin in visceral adipose tissue of diabetic rats are depicted in Figures 6, 7, and 8, respectively. The mRNA expressions of GLUT-4, insulin receptor *β*-subunit, and adiponectin mRNA expressions were significantly depleted in diabetic rats. The oral administration of *C. reticulata* fruit peel extract, hesperidin, and quercetin to diabetic rats significantly increased the suppressed mRNA expressions of GLUT-4 and insulin receptor *β*-subunit mRNA gene expressions. The treatment with hesperidin seemed to be the most potent in increasing adipose tissue insulin receptor *β*-subunit mRNA expression. Moreover, the effect of *C. reticulata* fruit peel extract and hesperidin produced a nonsignificant increase (*p* > 0.05; LSD) in visceral adipose tissue adiponectin mRNA expression relative to *β*-actin, while the treatment with quercetin induced a significant increase of adiponectin mRNA expression. Concerning one-way ANOVA, the effect between groups on visceral adipose tissue mRNA expressions of the detected genes was very highly significant since the *F*-probability has *p* < 0.001.

## 4. Discussion

T2DM is characterized by insulin resistance which may be due to insulin receptor and/or postreceptor defects. This defect leads to an impairment in the regulation of carbohydrate, lipid, and protein metabolism. Moreover, patients with T2DM have an increased hepatic glucose production and insulin insensitivity in skeletal muscle and adipose tissue or a combination of both and long-term persistent inflammation, all of which progressively disrupt control of glucose concentrations in blood and result in an occurrence of deleterious diabetic complications [72–74]. The persistent long-term hyperglycemia of DM is accompanied by chronic damage and dysfunction of different organs [75]. The NA/STZ-induced DM model has been proposed as experimental DM in rat since it exhibits features like human diabetes and is suitable for acute and chronic studies; thus, it is considered as a good model for the study of hyperglycemia [76–80].

STZ is often used to induce experimental DM in animals. The cytotoxic action of STZ is mediated by the excessive production of reactive oxygen species (ROS) causing oxidative damage that causes damage to *β*-cells *via* the activation of apoptosis and attenuation of insulin synthesis [11, 13, 81–83]. STZ enters the *β*-cell *via* GLUT-2, because it is similar enough to glucose so that it can be transported into the cell; however, it is not recognized by the other glucose transporter types (GLUTs). The *β*-cells possess high levels of GLUT-2; this explains the relative toxicity of STZ to *β*-cells [18]. The NA/STZ-induced T2DM rat model has been used in the present study because of the following lineaments: (a) stable moderate nonfasting hyperglycemia which requires no exogenous insulin to survive; (b) partial loss of *β*-cells in the islets of Langerhans (-40%); (c) decrease in islets' insulin content by 60%; (d) impaired glucose tolerance; (e) impaired glucose-stimulated insulin secretion; (f) responsiveness to sulfonylurea drugs; and (g) polyphagia and polydipsia [18].

Plants, used in traditional medicine to treat DM, represent valuable alternatives for the control of this disease [84]. The phytomedicines and functional foods play positive roles in the control and management of blood glucose concentrations, glucose uptake into peripheral tissues, insulin secretion, and immune function to prevent DM [85, 86]. Antioxidants from natural sources help in scavenging and removing ROS and significantly reducing the probabilities of progression of DM and its complications. Varieties of nutritionally important vitamins, some supplements, and ingredients from natural food sources may prevent the injury and deterioration caused by oxidative stress in DM. Hence, the plant kingdom has become an important target for the search of novel drugs and biologically active compounds [87, 88].

Tangerine (*C. reticulata*) fruit peel hydroethanolic extract contains rich phytochemicals as indicated in the present study by GC-MS and HPLC-MS analyses. Many of these phytochemicals have potent biological activities. 4H-pyran-4-one, detected by GC-MS at retention times of 16.556 and 27.356 minutes, is a cyclic nucleus in the chemical structures of many flavonoids including quercetin, naringin, hesperidin, nobiletin, and tangeretin. Polymethoxyflavones are represented in a GC-MS chromatogram at a retention time of 27.047 minutes. Moreover, the *β*-D-glucopyranose, 4-O-*β*-D-galactopyranosyl- peak observed at a retention time of 27.365 is a glycoside moiety of a number of flavonoids such as quercetin 3-*O*-*β*-D-glucopyranosyl(1→2)-*β*-D-galactopyranoside-7-*O*-*β*-D-glucuropyranoside (**1**) and kaempferol 3-*O*-*β*-D-glucopyranosyl(1→2)-*β*-D-galacto-pyranoside-7-*O*-*β*-D-glucuropyranoside [89]. On the other hand, HPLC-MS analysis indicated the presence of gallic acid, naringin, quercetin, hesperidin, nobiletin, and tangeretin. Hesperidin, naringin, and nobiletin were the most abundant. In the current study, hesperidin (which is a major component of the *C. reticulata* fruit peel hydroethanolic extract) and quercetin (which is one of its minor components) were purchased from Sigma-Aldrich Chemical Company (USA) and were tested for antidiabetic and antioxidant effects in comparison with the crude extract.

OGTT is a well accepted test to screen the antihyperglycemic efficacy of any hypoglycemic agents [1, 90, 91]. In the present study, NA/STZ-induced diabetic rats showed an elevation in serum glucose levels at all points of OGTT as compared with normal rats; these data are in concordance with other previous studies [92–95]. Concomitantly, the serum fructosamine level, which represents an accurate measure of mean blood glucose concentration over several days or weeks (2-3 weeks), was also significantly increased in NA/STZ-induced diabetic rats. Either a decrease in insulin secretion as in the case of insulin IDDM or a defect in tissue insulin sensitivity as in NIDDM could be the cause of a rise in serum glucose and fructosamine levels [96–98]. The elevation in blood glucose levels and the inhibition of the insulin synthesis may be referred to (1) a reduced uptake of glucose into peripheral tissues, muscles, and adipose tissues and insulin resistance which may not only induce hyperglycemia but also dyslipidemia [95, 99]; (2) a loss of activity and breakdown of the liver glycogen synthetase-activating system [100]; and (3) a significant decrease in the activities of the glycolytic and lipogenic enzymes, while there was an increase in the activities of gluconeogenic enzymes and hepatic glucose production [101].

The present study demonstrated that the treatment of diabetic rats with *C. reticulata* peel hydroethanolic extract, hesperidin, and quercetin caused potential ameliorative effects on OGT and the serum fructosamine level, and this is in concordance with the results of Jung et al. [102], Chakravarthy et al. [103], Chakravarthy et al. [104], Sharma et al. [105], Pandit et al. [106], and Kapoor and Kakkar [107] who revealed an analogous improvement effect of various flavonoids in different animal models of DM. An important finding of the present study is that the effect of *C. reticulata* fruit peel hydroethanolic extract has the most potent effect in improving OGT and in decreasing the elevated serum fructosamine level. The antihyperglycemic effect of the fruit peel hydroethanolic extract is more potent than the effects of hesperidin and quercetin due to the synergistic effects of these two flavonoids and other constituting flavonoids, such as naringin, and polymethoxylated flavones, *viz.*, nobiletin and tangeretin, as indicated by GC-MS and HPLC-MS analyses in the present study. These flavonoids have been reported to have antidiabetic effects by many investigators [1, 18, 84, 108].

Homeostatic model assessment (HOMA), estimated from fasting glucose and insulin levels, is a method used to calculate insulin resistance, insulin sensitivity, and *β*-cell function [109].

In the present study, HOMA-IR exhibited a significant increase in NA/STZ-induced diabetic rats while HOMA-IS and HOMA-*β* cell function showed a significant decrease; these changes in HOMA reflect the presence of insulin resistance (insulin insensitivity) and impaired *β*-cell function in the NA/STZ-induced diabetic rat model used in the present investigation. The treatment of diabetic rats with *C. reticulata* fruit peel extract, hesperidin, and quercetin successfully decreased elevated HOMA-IR and increased the lowered values of HOMA-IS and HOMA-*β* cell function. These ameliorations in the HOMA indices led us to suggest that all tested treatments produced antihyperglycemic and antidiabetic actions *via* improvements in both tissue insulin sensitivity and *β*-cell function. It is relative here to mention that the increase in serum levels of both insulin and C-peptide after treatment of diabetic rats with *C. reticulata* peel hydroethanolic extract, hesperidin, and quercetin, in the present study, supports the evidence that these treatments improved *β*-cell function and insulin secretory response. Moreover, in association with the improvements in serum insulin and C-peptide levels, the pancreatic islets exhibited an amendment of histological architecture and integrity together with the increase in the number of *β*-cells as a result of treatments of diabetic rats with *C. reticulata* peel hydroethanolic extract, hesperidin, and quercetin. On the other hand, the increase in adipose tissue GLUT-4 and insulin receptor-*β* subunit by treatments of diabetic rats with *C. reticulata* peel hydroethanolic extract, hesperidin, and quercetin reflects the improved adipose tissue (peripheral tissue) insulin sensitivity. Also, adiponectin significantly increased only as a result of quercetin treatment in the present study. As adiponectin is considered by previous publications as an insulin sensitizer [110, 111], it may importantly contribute to reduce insulin resistance and promote insulin-sensitizing activity due to the treatment of diabetic rats with quercetin.

Liver glycogen content may be regarded as biomarker for evaluating antihyperglycemic efficiency of any drug especially in experimental animals [95]. The raised hepatic glucose production in DM could be a result from glycogenolysis or gluconeogenesis or both, as stated by Ahmed et al. [112] and Raju et al. [101]. The present study exhibited a depletion in the hepatic glycogen content in association with an intense increase of hepatic glycogen phosphorylase and G-6-Pase activities when compared with normal rats. These results are in concordance with those of Ahmed et al. [112], Pari and Suman [84], Morral [113], and Ahmed [114], who demonstrated that NA/STZ-induced DM resulted in reduced hepatic glycogen content and elevated hepatic G-6-Pase and glycogen phosphorylase activities in diabetic rats. In the current study, the treatment of diabetic rats with *C. reticulata* fruit peel hydroethanolic extract, hesperidin, and quercetin induced a significant increase in liver glycogen content in association with a significant decrease in liver G-6-pase and glycogen phosphorylase activities; the three treatments have nearly similar effects. These improvements in the diabetic treated rats may reflect the decrease in hepatic glucose production which, in turn, leads to a decrease in the plasma glucose concentration. In addition to the previous mechanism, it was reported that the increased peripheral glucose uptake and reduced G-6-Pase activity may inhibit glycogen phosphorylase and also activate glycogen synthetase in the liver and muscles [115]. Furthermore, the present results also run parallel with Jung et al. [116] who revealed that hesperidin decreased the activities of gluconeogenic enzymes in T2DM. In addition to this, Eid and Haddad [117] stated that quercetin decreased G-6-Pase activity, which controls whole-body glucose homeostasis in NA/STZ-induced diabetic rats. In the same way, the current results are also in concordance with Mahmoud et al.'s study [1], which stated that flavonoids such as hesperidin and naringin caused a reduction in glycogen phosphorylase and G-6-Pase activities in NA/STZ-induced T2DM in rats. Thus, based on our results and those of previous publications, it can be elucidated that the improvements in the liver glycogen content and the decrease in glycogen phosphorylase and G-6-Pase activities as a result of treatments play an important role in the control of hepatic glucose production, and in turn, the control of blood glucose level. In addition, these ameliorations may be secondary to the increase in blood insulin level and alleviations in tissue insulin sensitivity.

It is well known that DM is usually accompanied by alterations in lipid and lipoprotein profile [91]. In the current study, the elevation in serum glucose level was concomitant with a remarkable increase in serum TC, LDL-C, and TG levels and a decrease in serum HDL-C in NA/STZ diabetic rats. These observations run parallel with Kapoor and Kakkar [107], Ulicna et al. [118], Wasan et al. [119], Wittenstein et al. [120], and Singh and Singh [121] who reported high levels of LDL-C and vLDL-C in poorly controlled diabetic patients and diabetic rats. In the present study, the treatments with the *C. reticulata* fruit peel hydroethanolic extract, hesperidin, and quercetin enhanced a significant alleviation of the altered serum lipids. These evidences are in accordance with the results published by Gorinstein et al. [122], Zhou et al. [123], and Li et al. [91], which demonstrated that the treatments with various flavonoids increased serum HDL-C and lowered the level of the TC, LDL-C, and TG in diabetic mice and diabetic rats. These ameliorative effects may be attributed to the evidence that flavonoids can be merged into lipoproteins and chylomicrons in both the intestine and the liver and subsequently be carried within these particles. Therefore, flavonoids could protect LDL-C from oxidation [124]. Thus, flavonoid consumption was inversely related to coronary heart disease (CHD) mortality [125]. In view of these findings from previous publications, our observations that the flavonoids hesperidin and quercetin have antihyperlipidemic effects are not new, but the new thing is that the *C. reticulata* fruit peel hydroethanolic extract has more potent improvement effects on serum TC and LDL-C levels than either of these two flavonoids. In our opinion, the higher effects of the fruit peel hydroethanolic extract may be due to the synergistic effects of multiple constituting flavonoids in the extract as indicated by the GC-MS and HPLC analyses.

In NA/STZ-injected rats, it was explained that insulin resistance, serum insulin level, and glucose intolerance may be attributed to a significant degree by changes in the serum FFA level [107, 126]. In the results of the current study, the diabetic rats exhibited an elevation in the serum FFA level. This elevation in the serum FFA level in diabetic rats was associated with the elevated value of HOMA-IR and the lowered values of HOMA-IS and HOMA-*β* cell function. Thus, there may be a relationship between the abnormal increase in FFAs and insulin resistance as well as *β*-cell function. In support of this elucidation, Oh et al. [127] reported that chronic elevated levels of FFAs resulted in insulin resistance and *β*-cell dysfunction; therefore, the decrease in the elevated plasma FFA levels may be an important therapeutic target in both obesity and T2DM. The long-term and persistent elevation in both blood glucose and FFAs leads to *β*-cell glucotoxicity and lipotoxicity that in turn results in damage of *β*-cells as well as impairments of the surviving *β*-cells' secretory response to the stimulatory effects of glucose and FFAs [127–129].

Upon treatment of diabetic rats with *C. reticulata* fruit peel extract, hesperidin, and quercetin, there was a decrease in serum FFA levels, which may be implicated to the stimulation of the insulin-sensitizing effects of treatment agents and may participate to improve the effects of the tested treatments on insulin secretory response and *β*-cell function. These results are in concurrence with Ahmed et al.'s publication [21] which declared that administration of *Citrus sinensis* fruit peel hydroethanolic extract and two of its constituting flavonoids naringin and naringenin to diabetic rats caused a significant decreased effect on the elevated serum FFA level concomitant with the improvements in the tissue insulin sensitivity and *β*-cell insulin secretory response. Thus, the decreasing effect of the tested treatments on FFA level, in the present study, is one of mode of actions of these treatments to enhance both insulin secretion and insulin sensitivity.

Hayes et al. [130] and Mossa et al. [131] revealed that one of the indicators for liver damage and dysfunction is the elevation in the activities of AST and ALT enzymes in the serum. These enzymes are used as biochemical indicators to evaluate the degree of liver dysfunction in STZ-induced diabetic rats. Consequently, the activities of liver damage markers of ALT and AST enzymes increased in the untreated diabetic patients [132, 133]. These data run in parallel with the results of Ahmed et al. [95] and Moneim et al. [134]. The *C. reticulata* peel hydroethanolic extract, hesperidin, and quercetin have a defensive effect against the hepatotoxicity produced by STZ-induced DM; their effects are nearly similar. In concurrence with the present study, Alam et al. [135] reported that liver functions were ameliorated in diabetic rats treated with hesperidin. Furthermore, Kobori et al. [136] reported an improvement of liver and pancreas functions by quercetin in STZ-induced diabetic mice.

The serum levels of creatinine and urea are considered as significant biomarkers of renal dysfunction which were increased in experimentally induced diabetes [137, 138]. Renal functions are affected by DM, and the long-term complications of the renal system are collectively known as diabetic nephropathy, which occurs due to the interactions of metabolic and haemodynamic factors [139]. The results in the present study had shown a significant elevation in serum creatinine and urea levels in diabetic rats when compared to a normal control group. These results run parallel with those of Yanardağ et al. [140], Yousef et al. [141], Elbe et al. [142], and Kandemir et al. [143]. Otherwise, the treatment of the present diabetic rats with *C. reticulata* fruit peel extract, hesperidin, and quercetin caused a significant decrease in creatinine and urea levels; hesperidin was the most potent. These evidences are in accordance with the finding of Elbe et al. [142], Kandemir et al. [143], and Abdel-Raheem et al. [144], who reported that serum creatinine and urea levels significantly decreased in STZ-induced diabetic rats treated with hesperidin and quercetin. In our opinion, the amendment of the deteriorated kidney function in NA/STZ-induced diabetic rats by treatments with *C. reticulata* fruit peel hydroethanolic extract, hesperidin, and quercetin may be secondary to the improvement of the diabetic conditions, suppression of oxidative stress, and enhancement of the antioxidant defense system.

Much attention has been paid to explore the role of oxidative stress, since it is widely believed that it contributes to the progress of DM and its complications [145]. Oxidative stress, which is an imbalance between formation and removal of highly reactive molecules, can lead to impaired insulin sensitivity, *β*-cells' dysfunction, impaired glucose tolerance, and eventually T2DM [146, 147]. In an oxidative stress condition, ROS are responsible for lipid and protein modifications on one hand, and the overproduction of ROS in DM is a direct consequence of hyperglycemia on the other [139, 148]. Authoritative with these findings, the current study displayed an increased MDA level and lowered activities of antioxidant enzymes, *viz.*, SOD, GPx, and GST in hepatic tissue of the STZ-induced diabetic rats [107, 147, 149–151]. The treatment of NA/STZ-induced diabetic rats in the current study with *C. reticulata* fruit peel extract, hesperidin, and quercetin produced a significant amelioration of liver levels of MDA, GSH, and antioxidant enzymes which are indicators of diminished oxidative stress *via* reversing the activities of these enzymatic antioxidants [150, 152, 153]. The effect of *C. reticulata* fruit peel extract on GPx activity was significantly more potent when compared with hesperidin and quercetin. These results are in accordance with Rajadurai and Prince [154] who revealed that flavonoids prevent alterations in mitochondrial lipid peroxides and enhance the activity of antioxidant enzymes (SOD, GPx, GSH, and GST) in rodents. Based on our results, it can be stated that the suppression of lipid peroxidation and enhancement of the antioxidant defense system as a result of treatments with *C. reticulata* fruit peel extract, hesperidin, and quercetin may play an important role in the amelioration of pancreatic islet histological integrity and function as well as tissue insulin sensitivity to improve the diabetic conditions in NA/STZ-induced diabetic rats.

## 5. Conclusion

In conclusion, the treatment with *C. reticulata* fruit peel hydroethanolic extract, hesperidin, and quercetin exerted antihyperglycemic and antihyperlipidemic effects which seemed to be mediated *via* enhancement of insulin release, insulin action, and antioxidant defense system in NA/STZ-induced diabetic rats. The improved insulin release was demonstrated *via* the increased serum insulin and C-peptide levels in addition to the elevated calculated HOMA *β*-cell function, while the improvement in insulin action was manifested by the increase in HOMA-IS and a decrease in HOMA-IR in association with the elevation in mRNA expression levels of insulin receptor *β*-subunit, GLUT-4, and adiponectin in adipose tissues. The *C. reticulata* fruit peel hydroethanolic extract has more potent antihyperglycemic and antihypercholesterolemic effects than hesperidin and quercetin. In addition to antihyperglycemic and antihyperlipidemic effects, *C. reticulata* fruit peel hydroethanolic extract, hesperidin, and quercetin have potent ameliorative effects on liver and kidney functions which may be secondary to the improvements in the glycemic state, lipid profile, and antioxidant defense system. In spite of these alleviative effects in NA/STZ-induced diabetic rats, further clinical studies are required to assess the efficacy and safety of *C. reticulata* fruit peel hydroethanolic extract, hesperidin, and quercetin in type 2 diabetic human beings.

## Figures and Tables

**Table 1:** Chemical composition of *C. reticulata* hydroethanolic extract as detected by GC-MS analysis.

Number	Retention time	Compound (from the central Library Search Report)	Area % (higher than 1%)
1	15.282	(i) No matches in library	3.50%
2	16.556	(i) 4H-Pyran-4-one, 2,3-dihydro-3,5-dihydroxy-6-methyl-	3.55%
3	17.906	(i) No matches in library	1.13%
4	18.080	(i) No matches in library	2.14%
5	18.579	(i) 5-Hydroxymethylfurfural (ii) 4-Hexen-3-one, 4,5-dimethyl-	17.74%
6	18.981	(i) No matches in library	1.57%
7	19.803	(i) No matches in library	2.65%
8	20.241	(i) No matches in library	1.84%
9	21.329	(i) Phenol, 4-ethyl- (ii) Benzaldehyde, 4-hydroxy- (iii) Benzaldehyde, 2-hydroxy-	1.00%
10	27.047	(i) 3,3′,4′,5,5′,7,8-heptamethoxyflavone	1.78%
11	27.356	(i) 4h-1-benzopyran-4-one, 2-(3,4-dimethoxyphenyl)-5,6,7-trimethoxy- (ii) *β*-D-Glucopyranose, 4-O-*β*-D-galactopyranosyl-	3.00
12	29.940	(i) Dodecane	1.29%
13	32.898	(i) n-Hexadecanoic acid (ii) Tridecanoic acid	2.88%
14	34.964	(i) 9,12-Octadecadienoic acid (Z,Z)-	2.37%
15	35.033	(i) 9,12,15-Octadecatrienoic acid, (Z,Z,Z)- (ii) (Z)6,(Z)9-Pentadecadien-1-ol (iii) 9,12,15-Octadecatrien-1-ol, (Z,Z,Z)-	2.46%
16	37.253	(i) 9-Octadecenamide, (Z)-	1.34%

**Table 2:** Serum insulin and C-peptide levels in normal, diabetic control, and diabetic groups treated with *C. reticulata* fruit peel extract, hesperidin, and quercetin.

Parameter groups	Insulin (ng/ml)	% change	C-peptide (ng/ml)	% change
Normal	1.93 ± 0.073^a^	—	2.98 ± 0.05^a^	—
Diabetic control	0.93 ± 0.04^c^	-51.81	0.99 ± 0.04^c^	-66.77
Diabetic treated with fruit peel extract	1.44 ± 0.10^b^	54.83	1.56 ± 0.10^b^	57.57
Diabetic treated with hesperidin	1.75 ± 0.05^b^	88.17	2.30 ± 0.13^b^	132.32
Diabetic treated with quercetin	1.53 ± 0.11^b^	62.51	2.29 ± 0.15^b^	131.31
*F*-probability	*p* < 0.001		*p* < 0.001	
LSD at 5% level	0.308		0.362	
LSD at 1% level	0.417		0.490	

Data are represented as mean ± SEM of six rats. Means which share different superscript letters are significantly different at *p* < 0.05. Percentage changes were calculated by comparing diabetic control with normal and diabetic treated groups with diabetic control.

**Table 3:** HOMA-IR, HOMA-IS, and HOMA-*β*-cell function in normal, diabetic control, and diabetic groups treated with *C. reticulata* fruit peel extract, hesperidin, and quercetin.

Parameter group	HOMA-IR	% change	HOMA-IS	% change	HOMA-*β*-cell function	% change
Normal	2.69 ± 0.16^c^	—	0.74 ± 0.02^a^	—	5.91 ± 0.37^a^	—
Diabetic control	5.35 ± 0.28^a^	98.88	0.34 ± 0.03^c^	-54.05	1.56 ± 0.17^c^	-73.60
Diabetic treated with fruit peel extract	3.31 ± 0.34^b^	-38.13	0.70 ± 0.03^b^	105.88	3.22 ± 0.25^b^	106.41
Diabetic treated with hesperidin	3.58 ± 0.28^b^	-33.08	0.62 ± 0.05^b^	82.35	3.91 ± 0.28^b^	150.64
Diabetic treated with quercetin	3.19 ± 0.23^b^	-40.37	0.64 ± 0.02^b^	88.23	3.08 ± 0.22^b^	97.43
*F*-probability	*p* < 0.001		*p* < 0.001		*p* < 0.001	
LSD at 5% level	0.73		0.10		0.84	
LSD at 1% level	0.98		0.12		1.14	

Data are represented as mean ± SEM of six rats. Means which have different superscript letters are significantly different at *p* < 0.05. Percentage changes were calculated by comparing diabetic control with normal and diabetic treated groups with diabetic control.

**Table 4:** Liver glycogen content, G-6-Pase, and glycogen phosphorylase activities in normal, diabetic control, and diabetic groups treated with *C. reticulata* fruit peel extract, hesperidin, and quercetin.

Parameter groups	Glycogen (mg/g tissue)	% change	G-6-Pase (mg Pi liberated/g tissue/hour)	% change	Glycogen phosphorylase activity (mg Pi liberated/g tissue/hr)	% change
Normal	22.02 ± 2.34^a^	—	18.89 ± 1.61^c^	—	12.35 ± 0.43^c^	—
Diabetic control	8.12 ± 0.80^c^	-63.12	59.92 ± 2.72^a^	217.20	25.97 ± 0.78^a^	11.02
Diabetic treated with fruit peel extract	14.03 ± 0.72^b^	72.78	35.30 ± 1.81^b^	-41.08	15.65 ± 0.047^b^	-39.73
Diabetic treated with hesperidin	15.81 ± 0.85^b^	94.70	36.10 ± 1.82^b^	-39.75	16.03 ± 0.67^b^	-38.27
Diabetic treated with quercetin	13.07 ± 0.50^b^	60.96	33.51 ± 2.31^b^	-44.07	15.70 ± 0.35^b^	-39.54
*F*-probability	*p* < 0.001		*p* < 0.001		*p* < 0.001	
LSD at 5% level	3.60		6.11		1.65	
LSD at 1% level	4.87		8.27		2.24	

Data are represented as mean ± SEM of six rats. Means which have different superscript letters are significantly different at *p* < 0.05. Percentage changes were calculated by comparing diabetic control with normal and diabetic treated groups with diabetic control.

**Table 5:** Serum lipid profile and FFA levels in normal, diabetic control, and diabetic groups treated with *C. reticulata* fruit peel extract, hesperidin, and quercetin.

Parameter groups	TC (mg/dl)	TG (mg/dl)	HDL-C (mg/dl)	LDL-C (mg/dl)	vLDL-C (mg/dl)	FFAs (mg/dl)
Normal	55.8 ± 2.23^c^	58 ± 3.10^c^	28.3 ± 0.73^a^	16.04 ± 1.87^c^	11.35 ± 0.63^c^	14.71 ± 0.33^c^
Diabetic control	92.43 ± 4.50^a^	99.36 ± 2.43^a^	18.94 ± 0.55^c^	52.70 ± 4.38^a^	19.95 ± 0.48^a^	36.43 ± 1.62^a^
Diabetic treated with fruit peel extract	65.65 ± 2.83^b^	77.48 ± 3.05	23.10 ± 0.56^b^	27.19 ± 3.16^b^	15.36 ± 0.53^b^	26.39 ± 0.93^b^
Diabetic treated with hesperidin	70.72 ± 2.79^b^	74.53 ± 3.59^b^	22.37 ± 0.44^b^	34.07 ± 2.40^b^	15.12 ± 0.69^b^	23.27 ± 0.62^b^
Diabetic treated with quercetin	73.37 ± 3.09^b^	78.96 ± 3.46^b^	22.88 ± 0.62	36.93 ± 3.72^b^	15.46 ± 0.53^b^	24.83 ± 1.15^b^
*F*-probability	*p* < 0.001	*p* < 0.001	*p* < 0.001	*p* < 0.001	*p* < 0.001	*p* < 0.001
LSD at the 5% level	9.27	9.19	1.72	9.43	1.70	3.01
LSD at the 1% level	12.55	12.44	2.33	12.77	2.30	4.07

Data are represented as mean ± SEM of six rats. Means which have different superscript letters are significantly different at *p* < 0.05. Percentage changes were calculated by comparing diabetic control with normal and diabetic treated groups with diabetic control.

**Table 6:** Serum AST and ALT activities in normal, diabetic control, and diabetic groups treated with *C. reticulata* fruit peel extract, hesperidin, and quercetin.

Parameter groups	AST (U/l)	% change	ALT (U/l)	% change
Normal	127.83 ± 2.22^c^	—	25.15 ± 1.14^c^	—
Diabetic control	193.33 ± 2.98^a^	51.23%	46.36 ± 1.45^a^	84.33%
Diabetic treated with fruit peel extract	148 ± 1.90^b^	-23.44%	31.00 ± 1.36^b^	-33.13%
Diabetic treated with hesperidin	131.5 ± 2.20^b^	-31.98%	33.06 ± 1.66^b^	-28.68%
Diabetic treated with quercetin	141.9 ± 3.16^b^	-26.60%	31.63 ± 1.42^b^	-31.77%
*F*-probability	*p* < 0.001		*p* < 0.001	
LSD at 5% level	8.98		5.15	
LSD at 1% level	12.15		6.97	

Data are represented as mean ± SEM of six rats. Means which have different superscript letters are significantly different at *p* < 0.05. Percentage changes were calculated by comparing diabetic control with normal and diabetic treated groups with diabetic control.

**Table 7:** Serum creatinine and urea level in normal, diabetic control, and diabetic groups treated with *C. reticulata* fruit peel extract, hesperidin, and quercetin.

Parameter groups	Creatinine (mg/dl)	% change	Urea (mg/dl)	% change
Normal	3.91 ± 0.49^d^	—	21.41 ± 1.9^d^	—
Diabetic control	8.45 ± 0.92^a^	116.11	35.28 ± 2.00^a^	64.78
Diabetic treated with fruit peel extract	6.03 ± 0.59^b^	-28.63	27 ± 2.01^b^	-23.46
Diabetic treated with hesperidin	4.98 ± 0.50^c^	-41.06	23 ± 1.80^cd^	-34.80
Diabetic treated with quercetin	5.40 ± 0.60^bc^	-36.09	25.31 ± 1.60^bc^	-28.25
*F*-probability	*p* < 0.001		*p* < 0.001	
LSD at 5% level	0.08		3.69	
LSD at 1% level	0.10		5.01	

Data are represented as mean ± SEM of six rats. Means which have different superscript letters are significantly different at *p* < 0.05. Percentage changes were calculated by comparing diabetic control with normal and diabetic treated groups with diabetic control.

**Table 8:** Liver oxidative stress and antioxidant defense markers in normal, diabetic control, and diabetic groups treated with *C. reticulata* fruit peel extract, hesperidin, and quercetin.

Parameter groups	LPO (nmole/MDA/100 mg tissue)	GSH (nmole/100 mg tissue)	GPx (mU/100 mg tissue)	GST (U/100 mg tissue)	SOD (U/mg tissue)
Normal	25.13 ± 0.68^c^	50.03 ± 2.6^a^	183.06 ± 1.7^a^	160.43 ± 1.73^a^	31.80 ± 1.97^a^
Diabetic control	56.82 ± 1.05^a^	21.13 ± 1.2^c^	113.97 ± 2.5^d^	83.11 ± 3.28^c^	17.43 ± 0.92^c^
Diabetic treated with fruit peel extract	39.09 ± 1.67^b^	32.33 ± 0.91^b^	153.66 ± 2.9^b^	116.94 ± 4.97^b^	24.98 ± 1.03^b^
Diabetic treated with hesperidin	36.39 ± 2.07^b^	32.70 ± 1.8^b^	136.54 ± 3.25^c^	112.77 ± 2.85^b^	23.75 ± 1.44^b^
Diabetic treated with quercetin	36.31 ± 2.16^b^	33.14 ± 1.9^b^	138.26 ± 2.60^c^	125.14 ± 4.18^b^	26.79 ± 1.57^b^
*F*-probability	*p* < 0.001	*p* < 0.001	*p* < 0.001	*p* < 0.001	*p* < 0.001
LSD at 5% level	4.765	5.35	9.31	10.44	4.39
LSD at 1% level	6.447	7.24	12.59	14.12	5.95

Data are represented as mean ± SEM of six rats. Means which have different superscript letters are significantly different at *p* < 0.05. Percentage changes were calculated by comparing diabetic control with normal and diabetic treated groups with diabetic control.

## Data Availability

The data used to support the findings of this study are available from the corresponding author upon reasonable request.

## Supplementary Materials

Supplementary Materials Schematic diagram that illustrates the effect of *Citrus reticulata* fruit peel hydroethanolic extract, hesperidin, and quercetin on NA/STZ-induced diabetic rats.
