# Supplementary figures and images for: Antidiabetic Potency, Antioxidant Effects, and Mode of Actions of Citrus reticulata Fruit Peel Hydroethanolic Extract, Hesperidin, and Quercetin in Nicotinamide/Streptozotocin-Induced Wistar Diabetic Rats
Source: Oxid Med Cell Longev. 2020 Jun 20;2020:1730492. doi: 10.1155/2020/1730492 (PMC7327566; doi:10.1155/2020/1730492)

Graphical abstract:

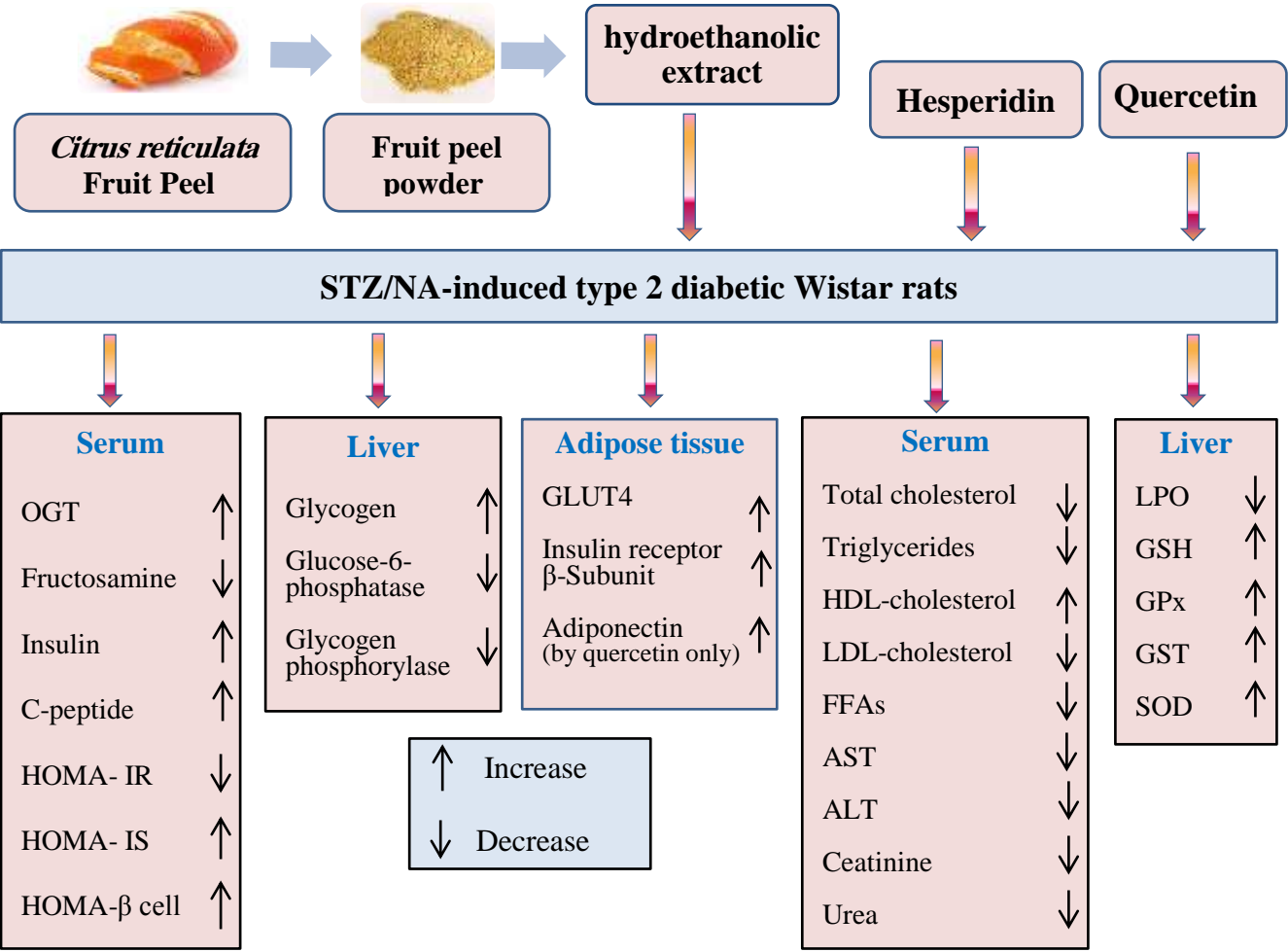

Supplement: Supplementary Materials — Schematic diagram that illustrates the effect of Citrus reticulata fruit peel hydroethanolic extract, hesperidin, and quercetin on NA/STZ-induced diabetic rats. [file 1730492.f1.pdf]
